# Resurrected *Ceriodaphnia quadrangula* highlight differences between pheno- and genotypic expressions

**DOI:** 10.1002/ece3.401

**Published:** 2012-11-02

**Authors:** Marko Reinikainen, Emma Åhlén

**Affiliations:** 1 Tvärminne Zoological StationJ.A. Palménin tie 260, FI-10900, Hanko, Finland; 2 Department of Ecology and Environmental Science, Umeå UniversitySE-901 87, Umeå, Sweden

**Keywords:** *Ceriodaphnia*, ephippia, fish, lake, palaeolimnology, perch, sediment

## Abstract

The hatching of cladoceran ephippia from a 15-cm long sediment core was investigated, and *Ceriodaphnia quadrangula* clones were isolated from different sediment layers. *Bosmina* microfossil data were also analyzed, and compared with the corresponding data from a Pb210 dated core, which allowed us to infer the age of the sediment layers. Using changes in *Bosmina* microfossil morphologies, we were, furthermore, able to infer the presence of different regimes of fish predation. *C. quadrangula* was found to hatch in layers with an inferred age of approximately a century. Newly hatched individuals had smaller eye-size in sediment layers corresponding to high predation by young-of-the-year perch. Newly hatched individuals also generally had a marked neck-spine. In contrast, morphological characters of *C. quadrangula* clones reared in the laboratory over several generations showed no variation in relation to predation regime, indicating the absence of fixed genotype level changes. Furthermore, the laboratory grown clones only rarely produced a neck-spine. The results suggest phenotypic variation in response to the regime under which ephippia were produced.

## Introduction

The term “resurrection ecology” has been used to describe an approach where long-lived dormant stages are utilized to study ecological changes over time periods that would be impossible to explore using observations on contemporary populations (Kerfoot et al. 1999). In plankton ecology, especially cladocerans and some copepods hatched from resting eggs have been successfully employed to study changes in life-history, behavioral, or genetic characteristics in response to varying selective forces (Brendonck and Meester 2003).

Cladocerans have a great advantage in experimental work, as parthenogenetic clones can be established. Most of the cladoceran studies have been carried out using *Daphnia*, which is abundant in many temperate lakes and also well-represented in ecological and evolutionary work in general. *Daphnia* has, for instance, been used to show adaptation in resistance to pollution (Kerfoot et al. 1999) and cyanobacterial toxins (Hairston et al. 1999). *Daphnia* has also been used to understand fitness consequences of hatching and reproduction success (Brede et al. 2007), to study changes in the strength of interspecific competition (Steiner et al. 2007), and to study the microevolution of diel vertical migration (Michels et al. 2007).

Typically, cladoceran ephippia have been reported to survive for a few decades (Brendonck and Meester 2003), which is substantially less than for certain species of copepods (Hairston et al. 1995). It is known, however, that *Daphnia* ephippia may be viable also for longer times, exceeding one century under some circumstances (Cáceres 1998; Michels et al. 2007).

While many cladoceran taxa other than *Daphnia* are also known to have egg banks (Moritz 1987; Brendonck and Meester 2003), these have been much less explored. As *Daphnia* may often occur in low numbers in lakes with heavy predation by fish, for instance in sub-arctic or boreal-forest lakes (Åhlen et al. 2011) or water bodies with increased salinity, compared with other taxa, the possibility to use these other taxa in “resurrection ecology” would allow the study of species that are perhaps in some instances more relevant as model organisms.

We also note that most of the previous study has focused on “before–after” approaches, that is, a population covering a time period before a certain event has been compared with a population covering a later period. The information provided from these studies has been extremely valuable, and the methods have been appropriate for the hypotheses tested. Nevertheless, it has also been shown (Michels et al. 2007) that it is possible to reconstruct a finer timeline of clonal succession. This requires, however, methods to reconstruct changes in evolutionary pressures, an egg-bank that is rich enough for extraction of eggs with a reasonable effort, and an appropriate resolution in the sediment in relation to egg viability and the environmental variable studied.

If the above requirements are met, cladoceran ephippia could provide an excellent model to study not only major differences in characteristics produced under different regimes, but also rates at which differences are manifested in the highly variable environments cladocerans often experience.

Moreover, the cladoceran egg-bank can serve as a source to study not only the (fixed) genotypic manifestations, but also the phenotypic variation in the wild-type populations that are produced under changing environmental conditions. Although the production of new genotypes in evolutionary processes remains unchallenged in ultimately causing diversification in organisms, the role of phenotypic plasticity should not be ignored in studying the response to environmental change. As phenotypic plasticity may provide an alternative to the selection for genotypes with fixed properties, and as phenotypic plasticity may in fact be an adaptive trait in itself (Price et al. 2003; Svanbäck et al. 2009), failure to record phenotypic expressions may mask microevolutionary processes other than selection for genetically fixed traits. For instance, Michels et al. (2007) show that the diel vertical migration pattern is similar for *Daphnia* from different predation-regimes when inductive signals from fish (kairomones) are absent, whereas clones from regimes with high fish predation migrate more actively in the presence of these signals.

In addition to these motives to consider phenotypic plasticity, it is also important to note that the role of phenotypic plasticity in channeling genetic diversification has received considerable attention in the recent literature (Snell-Rood et al. 2010). The mechanisms (reviewed and defined in Crispo 2007) include changes in mean trait values within plastic responses (the Baldwin effect; plasticity influences the survival of an individual in a new environment, dictating the course of further evolution), or driving new, genetically fixed traits through actions on developmental systems involved in producing different phenotypes (genetic assimilation). “Resurrection ecology” at different time-scales and especially with a fine-tuned timeline could provide important insights into the operation of these processes in nature, if both fixed genotypic expressions and the plasticity expressed in wild-type populations are considered.

In this study, we investigate the viability of ephippia from *Ceriodaphnia quadrangula* in the sediment of a boreal-forest lake. We use *C. quadrangula* to test the hypothesis that eye-size decreases as a microevolutionary response to visual predation (smaller eyes are less visible to fish; Branstrator and Holl 2000), and similarly, that mucro-length (the length of the tail-spine) decreases (Kerfoot 1981; Sweetman and Finney 2003). Furthermore, we explore the occurrence of neck-spines; antipredatory characteristics known to vary geno- and phenotypically in cladocerans (Lass and Spaak 2003). We explore these characteristics in newly hatched individuals, which reflect phenotypic differences in the ephippia produced under different conditions. By also using individuals reared in the laboratory over several generations, we furthermore explore variation at the genotype level under different historic conditions with regard to the above traits.

It is important to note that to date our understanding of planktivory in the study lake is restricted to fish, as invertebrate predators (dominated by *Bytotrephes)* have not been monitored. This sets limitations especially on the interpretation of neck-spines, which occur as defense against invertebrate predation (reviewed by Lass and Spaak 2003). In pilot studies preceding this experiment it was, however, noted that there was marked variation on the size and occurrence of neck-spines, and we decided to include this variable to study its plasticity versus genotype level manifestations. Hence, our interpretations on neck-spine production are limited to this aim, whereas the selective forces operating on the individuals present in the egg-bank remain to be investigated.

We tentatively infer the age and fish-predation regime of each sediment layer from a previously established calibration curve based on paleolimnological data on *Bosmina* morphology. The calibration curve was created from an independent, Pb210-dated core, using the information from a training set of 39 lakes where the effects size and species specific effects of fish predation on *Bosmina* morphology were investigated (Åhlen et al. 2011).

Since 1992, the study lake has been intensively monitored, and hence for the more contemporary sediment layers, we back up the findings in the *Bosmina* record with known changes in planktivory (Persson et al. 2004a). For historic changes, however, the inference remains more hypothetic, as it relies on the changes in *Bosmina* morphology, anecdotal information on fish introductions, and observations from neighboring lakes (namely dominance by ninespined stickleback in lakes with no fish introductions). Data on invertebrate predation are lacking, and therefore the role of these predators in forming the morphological characteristics studied remains to be solved in future investigations.

## Methods

### Study lake

The study was conducted on sediment samples obtained from Lake Abbortjärn 3, a small oligotrophic lake located in Åmsele, central Sweden (64°29′ N, 19°26′ E) (Persson et al. 1996). The lake has been used extensively in studies on fish predation–prey dynamics for almost two decades, and also in recent paleolimnological work, focusing on reconstructing fish predation regime (Åhlen et al. 2011). Today, the only fish species present in the lake is perch. Planktivory is high in the lake when there are high numbers of recruits; alternative phases when planktivory is low appears when the population is dominated by stunted cannibals (Persson et al. 2004a). Table 1 shows how recruitment young-of-the-year (YOY) perch varied between 1992 and 2000. Historic information on the lake is scarcer. It is known that neighboring lakes (less than 500 m) that have not been subject to artificial fish introductions are inhabited by ninespined stickleback, and characterized by a constant high planktivory by fish (Johansson and Wahlström 2002). In the present study lake, an inventory was made in 1957 by the forest company that owns the lake, and the presence of ninespined stickleback and perch was documented. A likely scenario is that introduction of perch gradually led to the disappearance of ninespined stickleback, as perch today is the only fish species present, and as local fishery observations suggest that this has been the case since the beginning of the 1970s (Greger Almerson, pers. comm.).

**Table 1:** The densities of young-of-the-year (YOY) perch in Lake Abbortjärn 3 in 1992–2000

Year	YOY perch
1992	288
1993	403
1994	28,800
1995	3072
1996	7488
1997	4857
1998	4550
1999	288
2000	269

The density of YOY perch were calculated from the spring densities of 1-year perch (Persson et al. 2004a) and the estimated average survival of YOY perch in the lake over the winter (0.104; Huss et al. 2008).

### Sediment sampling and treatment

The sediment samples for the experiment were obtained from a core taken at 10-m depth, in a ca 0.15-ha basin of the lake. A HTH-gravity corer (HTH-Teknik, Vårvägen 37, SE-976 31 Luleå, Sweden) was used. The samples were sliced on location into 0.5-cm slices from the sediment depth 0–15 cm, and transported to the laboratory where they were stored for 1 week at 4°C in the dark. After that, the samples were weighed, and a ca 1-g subsample was obtained from each slice, to be used for later paleolimnological analysis of cladoceran remains. The rest of each sample was placed in individual 900-mL, semitransparent plastic jars, which were filled with 50-μm sieved lake water and loosely covered with a lid. The lake water hence contained phytoplankton and bacteria, whereas other zooplanktons were removed. The lake water was aged for ca 1 month prior to the start of the experiment. Our pilot studies revealed that seminatural lake water was superior in promoting survival of the hatchlings compared with either filtered water or artificial water (see also below – preparation of culture water).

The jars were placed in random order in a climate chamber adjusted to a temperature of 12°C, and a light regime of 17:7 h L:D. The jars were checked weekly until hatching ceased (ca 1 month) by pouring the contents of each jar through a 50-μm sieve, the contents of which was rinsed into a Petri dish, from which hatched individuals were removed. Depending on the amount of individuals present, a maximum of 15 *Ceriodaphnia* specimens from each sediment depth were transferred individually to 300-mL culture bottles – arranged randomly – for further culture (see below). The rest of the hatched individuals were preserved in 70% ethanol for further identification and measurements; these animals represented the newly hatched generation.

After all the clones had been isolated, they were reared for 10 weeks in the laboratory. Our pilot studies indicated that under our culture conditions this procedure allowed for the production of 7–10 generations. Hence, in 10 weeks, we assume that maternal effects deriving from the differing lake conditions under which the ephippia had originally been produced were minimized. The water for the cultures was prepared by placing ca 2-L of lake sediment, collected from 0 to 10 cm sediment depth, into plastic containers. The openings of the containers were tightly covered with a 50-μm mesh, after which they were submerged in ca 70-L of sieved lake water for 1 week. This allowed a seminatural community of phytoplankton and micro-organisms to develop in the lake water. Our pilot studies revealed that this culture water was highly superior to standard laboratory rearing methods (e.g., monocultured algae) in minimizing laboratory selection among clones, promoting reproduction, and diminishing ephippial production. Prior to use, the culture water was passed once more through a 50-μm mesh. The water in the culture bottles was renewed weekly with fresh water, prepared as described above. The rearing of the *Ceriodaphnia* clones took place under the same conditions as the hatching of the eggs. After 10 weeks, *Ceriodaphnia* individuals for morphological measurements were obtained by placing egg-bearing females in freshly prepared culture bottles, which were sampled for juveniles to be used in the analyses. When possible, 3–5 juveniles per clone were used to produce average clone values used in statistical analyses.

### Morphological measurements

After termination of the experiment, we measured mucro-, carapace-, and neck-spine-length, as well as eye-size in both the newly hatched generation and in the laboratory grown clones. As the hatching frequency decreased with increasing sediment depth, animals from the newly hatched generation were available only down to 4.5-cm sediment depth (approximate year 1983). Under that sediment level, the number of hatchlings was below 15 (number aimed to achieve in rearing experiment), and hence no animals from older layers than 1983 were available for the measurements of the newly hatched generation. Nevertheless, 88 animals were available for analysis of the newly hatched generation. From the rearing experiment, a total of 117 clones, and 342 individuals, were available for the analyses.

The morphological characters were measured using an Olympus inverted light microscope at 100–200× magnification for mucro- and carapace-length, and 600× magnification for eye-size. Both the height and width of the eyes were measured, and an ellipsoid shape was assumed for calculation of eye-area.

We also analyzed the remains of *Bosmina* spp. from the subsamples obtained from each sediment layer in both experiments. This was carried out by heating the wet sediment on a hot plate in 25 mL 10% KOH at ca 70°C for 30–60 min and sieving it through a 100-μm sieve. The residuum was stored in 100-mL of water and acid lugol. The remains were counted and measured at 100 or 200× magnification using an Olympus inverted light microscope. From each sample, the carapace- and mucro-lengths from 20 remains were measured.

**Figure 1:**
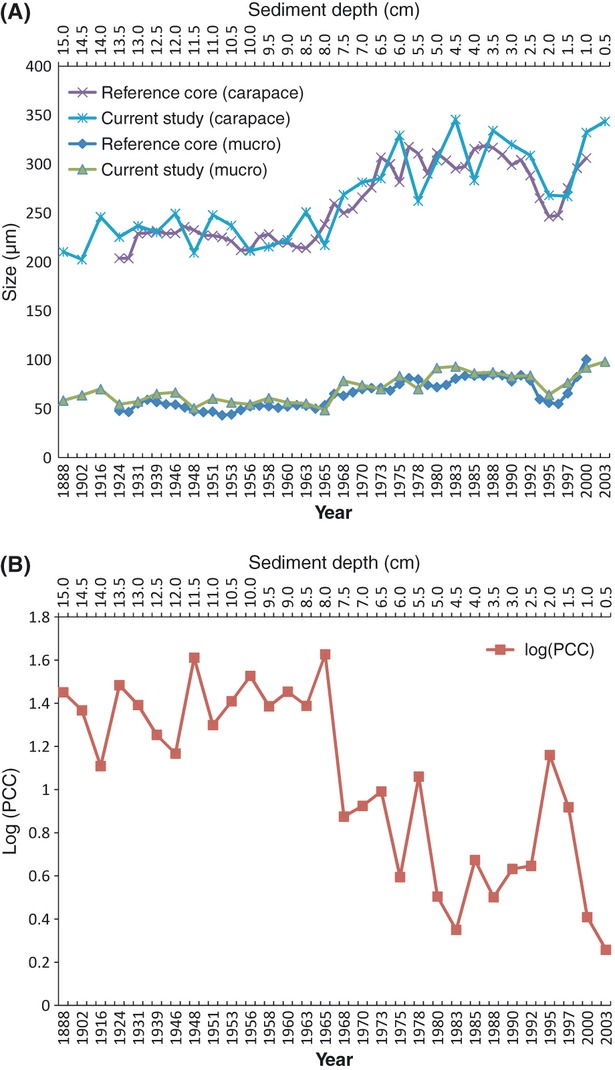
(A) Mucro- and carapace-length in *Bosmina* microfossils collected from two sediment cores. The reference core is a Pb210-dated core. (B) Predation intensity (log[PCC]) calculated from the mucro- and carapace-data in the current study. In both panels, the year corresponding to each data-point in the reference core is given on the primary (lower) *x*-axis. The depth corresponding to each data-point in the core used for the current study is shown on the secondary (higher) *x*-axis (each number indicates lower edge of respective sediment layer).

The *Bosmina* data derived from the sediment core was used to identify major changes in fish predation regime, and as an indirect way to approximate sediment age. This indirect dating approach is based on a previously established relationship between fish predation and mucro- and carapace-length in *Bosmina* morphology (Åhlen et al. 2011). According to this method, the planktivore community capacity (PCC) can be calculated using values for mucro- and carapace-length of *Bosmina*, together with lake area (see Åhlen et al. 2011 for equation and details). Using the sediment from a Pb210-dated core (reference core), taken within the same 10-m depth lake-basin (Fig. 1A; E. Åhlén et al., unpubl. data), we attempt to identify the changes in predation that are known to have occurred (Table 1), in the *Bosmina* data. By aligning the *Bosmina* data from the Pb210-dated reference core and from the core that the *Ceriodaphnia* were isolated from, and by identifying the same indicators of known changes in fish predation, we hence attempt to also infer the corresponding ages in the nondated core.

### Statistical analyses

The morphological data of *Ceriodaphnia* were analyzed using analysis of covariance (ANCOVA), where eye-area, mucro-length, and neck-spine-height were used as dependent variables, and carapace-length was used as a covariate. The covariate was used, as we were interested in the relative changes of these values, rather than the absolute values. For the individuals from the rearing experiment, we calculated a mean value for each variable and clone. Hence, each clone represented a replicate for its sediment depth in the ANCOVA. The individuals isolated directly upon hatching were, however, viewed as independent samples of animals emerging from the egg-bank, and were treated as individual replicates. SAS 9.2 (GLM-procedure) was used in the analyses.

## Results

### Inferred fish predation regime and sediment age

The paleolimnological data on *Bosmina* morphology revealed a distinct dip in the mucro- and carapace-lengths around 2-cm sediment depth, and a clear decrease in these measures from 8-cm sediment depth downward (Fig. 1A). The profiles were highly similar to previously reconstructed profiles based on a dated sediment core (E. Åhlén et al., unpubl. data). Correlations between the current data and the corresponding measures in the dated core were highly significant (Pearson's r for mucro- and carapace-lengths were 0.89 and 0.86, respectively; *P* < 0.001 in both cases). The data from the dated sediment core also established a relationship between fish predation intensity and *Bosmina* microfossil characteristics in the same basin of the lake.

An approximation of the age of the sediment layers can hence be made by aligning the previously established curve with the present one, and two high-predation regimes can be suggested. First, the response in *Bosmina* morphology around 2-cm sediment depth agrees well with the observed high prevalence of YOY perch between 1994 and 1998 (Table 1). Second – although much more speculatively – the *Bosmina* profile from ca 8-cm downward is also in agreement with the suggested presence of ninespined stickleback (*Pungitus pungitus*) before the establishment of perch in the 1960s. Unfortunately, there are no scientific data to further confirm this hypothesis; only local fishery observations (Greger Almerson, pers. comm.) together with the fact that neighboring lakes (<500 m) that have not been subject to introductions of other fish host strong populations of stickleback (Johansson and Wahlström 2002).

Figure 1B also shows the predation intensity calculated from the present data (Åhlen et al. 2011). We here used a recently derived approach to estimate past planktivore pressure in lakes, PCC (Åhlen et al. 2011). PCC takes into account the size and species specific foraging efficiencies of fish on zooplankton. PCC was obtained by summing the attack rates for each individual fish and dividing the sum of the attack rates of all fish by number of gill nets. In this way, a fish community attack rate per unit effort was obtained.

### Hatching profile

*Ceriodaphnia quadrangula* was found to hatch in large numbers down to inferred sediment ages of ca 20 years (5-cm depth), but also hatched frequently in the older layers. The oldest sediment layers where eggs could be hatched had an inferred age of ca 115 years (15-cm sediment depth). The hatching profile of *C. quadrangula* together with other cladocerans (*Bosmina* spp. and *Holopedium gibberum*) is shown in Appendix.

### Morphological characters

**Figure 2:**
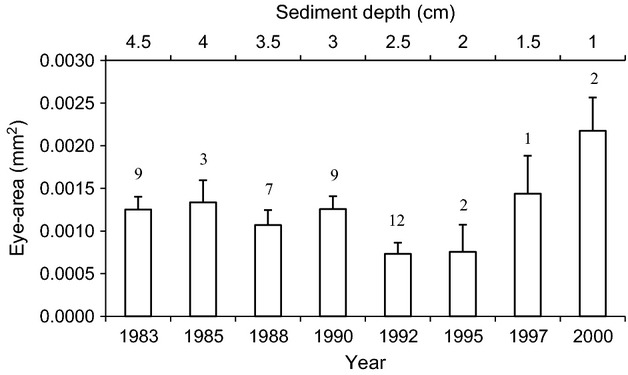
Marginal means (from ANCOVA; adjusted for carapace-length) + standard error of the eye-area in *Ceriodaphnia quadrangula* clones isolated from different sediment depths, and measured upon hatching. The inferred year corresponding to each sediment layer is given on the primary (lower) *x*-axis. The sediment depth from which the clones were hatched is given on the secondary (higher) *x*-axis (each number indicates lower edge of respective sediment layer).

**Figure 3:**
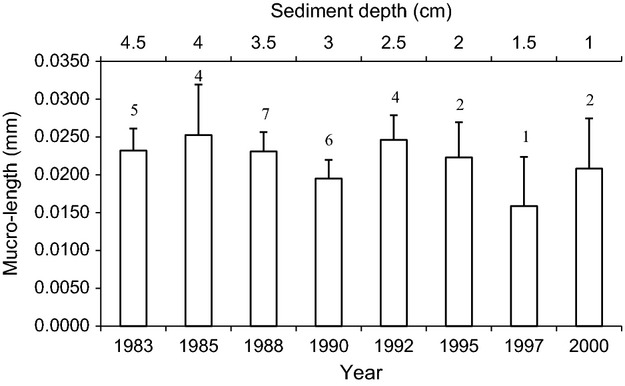
Marginal means (from ANCOVA; adjusted for carapace-length) + standard error of the mucro-length in *Ceriodaphnia quadrangula* clones isolated from different sediment depths, and measured upon hatching. The inferred year corresponding to each sediment layer is given on the primary (lower) *x*-axis. The sediment depth from which the clones were hatched is given on the secondary (higher) *x*-axis (each number indicates lower edge of respective sediment layer).

**Figure 4:**
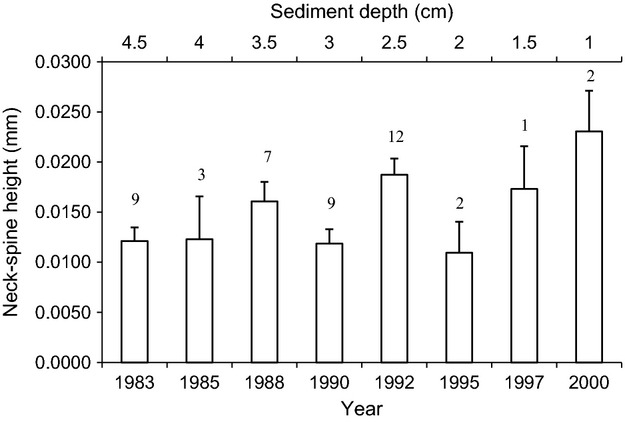
Marginal means (from ANCOVA; adjusted for carapace-length) + standard error of the neck-spine height in *Ceriodaphnia quadrangula* clones isolated from different sediment depths, and measured upon hatching. The inferred year corresponding to each sediment layer is given on the primary (lower) *x*-axis. The sediment depth from which the clones were hatched is given on the secondary (higher) *x*-axis.

For *C. quadrangula* individuals preserved directly upon hatching (newly hatched generation), a statistically significant difference between the sediment depths was observed for eye-area, which was smallest in individuals from the 2–2.5 cm sediment layers; individuals in the top-most layer had the largest eye-area (Fig. 2; ANCOVA *df* = 7; MS = 6.35 × 10^−7^; *F* = 3.20; *P* < 0.001). The covariate (carapace-length) was also statistically significant (*df* = 1; MS = 1.21 × 10^−6^; *P* < 0.02). For mucro-length, no similar effect was observed (Fig. 3). A neck-spine was present in 74% of the newly hatched generation. Neck-spine-height differed significantly among the sediment depths (ANCOVA *df* = 7; MS = 5.1 × 10^−5^; *P* < 0.03), but no clear-cut trends in relation to changes in fish predation could be identified (Fig. 4). The covariate (carapace-length) did not have a statistically significant effect on the neck-spine height.

**Figure 5:**
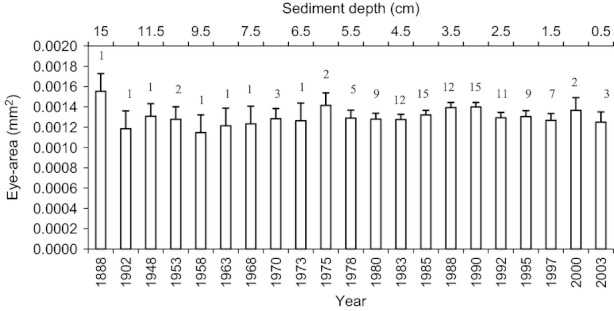
Marginal means (from ANCOVA; adjusted for carapace-length) + standard error of the eye-area in *Ceriodaphnia quadrangula* clones isolated from different sediment depths, and reared in the laboratory over several generations. The inferred year corresponding to each sediment layer is given on the primary (lower) *x*-axis. The sediment depth from which the clones were hatched is given on the secondary (higher) *x*-axis (each number indicates lower edge of respective sediment layer).

**Figure 6:**
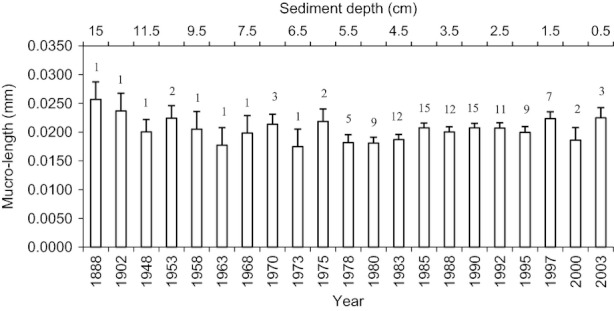
Marginal means (from ANCOVA; adjusted for carapace-length) + standard error of the mucro-length in *Ceriodaphnia quadrangula* clones isolated from different sediment depths, and reared in the laboratory over several generations. The inferred year corresponding to each sediment layer is given on the primary (lower) *x*-axis. The sediment depth from which the clones were hatched is given on the secondary (higher) *x*-axis. (lower) *x*-axis (each number indicates lower edge of respective sediment layer).

Neither the eye-area (Fig. 5) nor the mucro-length (Fig. 6) measured from the isolated and laboratory grown *C. quadrangula* clones differed significantly among the different sediment depths (ANCOVA). Neck-spines were present only in 10.2% of the animals, and could not be analyzed statistically, as *n* was too low.

## Discussion

This study is to the first one to explore the egg-bank of other cladocerans than *Daphnia* in relation to sediment age and lake trophic dynamics (but see Moritz 1987). In our study, an indirect dating approach was used by comparing the profiles of *Bosmina* morphology in a Pb210 analyzed core to a similar profile attained from the core that was used to hatch the cladocerans presented here. Thus, the sediment age should clearly be viewed as tentative. Nevertheless, the *Bosmina* profiles from the two cores fitted remarkably well, and two separate and major changes in fish predation, which have been historically documented in the lake, could be identified in both cores. We suggest therefore that the isolated *C. quadrangula* individuals can be viewed as representatives for different predation-regimes.

As indicated by the *Bosmina* profile, it is highly likely that the oldest *C. quadrangula* clones represent early 20th century or late 19th century animals, suggesting that *Ceriodaphnia* ephippia may survive at least as long as *Daphnia* (Cáceres 1998; Michels et al. 2007), and also markedly longer than previously suggested (Moritz 1987).

In this study, we made use of the relatively rich *C. quadrangula* egg-bank to study the variation in certain morphological traits (mucro-length, neck-spine-height, eye-area) suggested from earlier work to covary with predation (Kerfoot et al. 1999; Branstrator and Holl 2000; Sweetman and Finney 2003). In the clones reared in the laboratory over several generations, there was no statistically significant variation among the different sediment layers. Hence, the different predation-regimes, known to have occurred in the lake, have not selected for genotypes that would express constitutive differences in these traits. In fact, one of the traits (neck-spine) was virtually missing altogether in the laboratory population.

The animals preserved upon hatching, representing the first exephippial population, differed, however, from the laboratory population in two ways. First, they showed variation in eye-area, and secondly they typically had conspicuous neck-spines. These differences among the two populations suggest phenotypic plasticity in eye-size and neck-spine production.

Eye-area was smallest around the inferred peak in fish predation, being ca 50% smaller than the average of the other exephippial clones, and indeed than in the laboratory grown clones from the same depths. At first sight, the match was not perfect as the decreased eye-size appears already slightly before the onset of strong perch recruitment, and the effect was again relaxed before planktivory decreased. However, the resolution in the layers is not annual – for instance, the layer with the tentative date 1992 at the lower edge theoretically contains sediment from 1992 to 1994 (the latter year representing the onset of high planktivory). In reality, therefore, the layers are likely to contain a mix of clones from different regimes. Also, admittedly, the aligning of the study core and the reference core could cause dating errors as the two profiles are not identical, although show a high degree of similarity.

Keeping these uncertainties in mind, we suggest that we were able to roughly pinpoint a sediment region with a presence of clones from extremely high planktivory by YOY perch, favoring smaller eye-size. The compound eye is believed to be an important cue for visually oriented planktivorous fish (Branstrator and Holl 2000). However, the compound eye is of vital importance in cladocerans for phototaxis (e.g., diel vertical migration) and orientation of the body axis during swimming movements (Ringelberg 1999). Thus, a trade-off between optimal eye-size for swimming behavior and visibility to predators seems likely. Our results suggest that fish predation may select for smaller eye-size, but that the mechanism is phenotypic plasticity, rather than selection for genotypes with consistently smaller eyes. This kind of plasticity would allow the animals to produce larger eyes in the absence of predators, whereas (in this case ephippial) offspring produced under high-predation -regimes are born with smaller eyes. The signal for the induction of smaller eyes cannot, unfortunately, be explored here, but the presence of fish kairomones is a plausible explanation of the induction.

The production of neck-spines is an extremely well-studied example of a plastic antipredatory trait in cladocerans; especially *Daphnia* has been used as a model organism in numerous studies (reviewed in Lass and Spaak 2003), where the production of neck-spines especially in the presence of the phantom midge *Chaoborus* has been demonstrated (Spitze 1992; Tollrian 1993). Induction of neck-spines lowers the predation risk from the gape-size limited predators (Krueger and Dodson 1981; Havel and Dodson 1984). The production of neck-spines is not, however, typically an efficient defense against larger predators, such as fish. Therefore, fish kairomones are generally believed to induce other kinds of morphological or behavioral responses (Boeing et al. 2006). Hence, it is not surprising that in the present study, the variation in neck-spine height in the ephippial generation, although statistically significant, could not be related to differences is fish predation regime (Fig. 4). (Other factors, such as variation in invertebrate predators, cannot be explored here.) It is, nevertheless, interesting to note that the vast majority (74%) of the newly hatched generation had neck-spines, whereas this trait was rare (10% of the animals) in the laboratory grown clones.

In *Daphnia*, it is known that induced neck-spines can be passed on for a few generations, after which it disappears if predators are absent (Agrawal et al. 1999). Our study shows that most of the animals that hatch from the sediment are born with a neck-spine, but that the trait virtually disappears after several generations in the laboratory. It is possible that the occurrence of neck-spines is due to induction from predator cues in the parental generation. It is, however, also possible that ephippial animals are typically born with a neck-spine in this lake even without chemical induction, as a “safe” strategy to meet with an unpredictable predator environment. The difference between these mechanism cannot be elucidated here, but it is clear that *Ceriodaphnia* shows strong plasticity in this trait.

As already stated, neck-spine production, as well as other morphological defenses, is well-studied in *Daphnia*, as reviewed by Lass and Spaak (2003). It is likewise well known that the production of these defenses is typically plastic, and requires induction by info chemicals – without the presence of these chemicals, the traits gradually disappear. Most studies have been conducted on contemporary animals, isolated from watersheds and reared in the laboratory in different treatments. Here, we show that the egg-bank provides a valuable tool for the study of phenotypic plasticity over historic scales. It should be noted, however, that whereas egg banks have the potential to study microevolution temporally over relatively long time periods, the trade-off is that seasonal succession is difficult – possibly even impossible – to include in egg-bank studies, as it would require extremely high resolution. Seasonal succession of clones with different responses to predators has for instance been studied by Stibor and Lampert (2000). We suggest, nevertheless, that adding egg-bank studies to the tool-box will provide important insights into, for example, the relative importance of seasonal processes and longer term historic changes.

Phenotypic plasticity can be viewed as an adaptive trait favored in fine-graded environments, that is, habitats where the organism may encounter exhibit a high degree of variability. In contrast, coarse-graded environments exhibit predictability, and are expected to decrease the level of adaptive plasticity (Hollander 2008). In this study, the predation-pressure caused by YOY perch since the 1960s shows remarkable heterogeneity due to variation in perch recruitment (Fig. 1B; Persson et al. 2004b), and can be viewed as a fine-graded environment for the zooplankton prey. Maintenance – and even promotion (Svanbäck et al. 2009) – of plasticity can hence be viewed as adaptive, consistent with the expectations in a variable environment. In contrast, the predation regime before the introduction of perch was – possibly – characterized by a consistent high planktivorous pressure, as suggested by our sediment data (Fig. 1B) and the hypothesized presence of ninespined stickleback before introduction of perch. If so, then despite a high-predation regime lasting for several decades, the laboratory grown clones exhibited highly similar eye-sizes, indicating no genetically constitutive differences. This observation contrasts with the notion that a course-graded environment should genetically shift mean trait values. However, as pointed out by (Hollander 2008), the grain-size concept is affected also by the likelihood of gene flow between habitats, that is, by the probability of dispersal. Cladoceran ephippia (as evidenced in studies on *Daphnia*) are believed to be transported between water bodies by waterfowl and other animals by several mechanisms, including gut-passage or external attachment to, for example, feathers (Slusarczyk and Pietrzak 2008 and references therein). Maintained plastic morphological responses due to dispersal factors have also been shown, for example, in studies on freshwater snails from different predation-regimes (Brönmark et al. 2011). Our study lake is located in the close vicinity of several other lakes with varying historic fish-regimes (Persson et al. 2004b), providing possibilities for dispersal, and suggesting that the *Ceriodaphnia* populations are, in fact, facing a fine-graded environment promoting phenotypic plasticity.

Our study hence demonstrates variation in the newly hatched generation in some morphological traits, and the variation in eye-area is in accordance with the hypothesis of reduced eye-size when visually oriented predators are abundant. The response was rapid, and occurred within the dynamic changes in recruitment success of perch in the lake (Persson et al. 2004a,b). Our study furthermore shows differences between the newly hatched generation and the laboratory grown clones, suggesting phenotypic plasticity. We were not, however, able to show that different predation-regimes produced genotypes that consistently differed in their morphology. It is possible that life-history studies would provide a more powerful tool to find differences also at a fixed genotype level. Fish predation is known to affect a number of traits, including growth, timing of reproduction, clutch-size, and offspring-size (Lass and Spaak 2003). The rich egg-bank of *C. quadrangula* also provides an excellent tool for life-history studies, and as has been shown here, the *Bosmina* microfossils can be used to reconstruct changes in fish predation regime.

## Appendix

Number of individuals of four cladoceran species hatching from different sediment depths. The inferred year corresponding to each sediment layer is given on the primary (lower) *x*-axis. The sediment depth from which the ephippia were hatched is given on the secondary (higher) *x*-axis (each number indicates lower edge of respective sediment layer).
